# Nerve-Sparing Parotidectomy Guided by Nerve Auto-Fluorescence Technology

**DOI:** 10.7759/cureus.77025

**Published:** 2025-01-06

**Authors:** Fernando Dip, Rene Aleman, Alberto Rancati, Raul J Rosenthal, Diego Sinagra

**Affiliations:** 1 Department of General Surgery, University of Buenos Aires, Buenos Aires, ARG

**Keywords:** minimally invasive surgery, nerve auto-fluorescence technology, nerve-sparing surgery, parotidectomy, parotid gland disease

## Abstract

Parotidectomy is a commonly performed surgery for various indications, including inflammatory conditions, infection, congenital symptomatic malformations, and neoplasm resection. Irrespective of its indication, the performance requires meticulous surgical navigation by highly experienced surgeons because of its proximity to the facial nerve. While the surgical technique continues to evolve, nerve paralysis and nerve-related complications remain a significant concern following an intervention. Due to the high learning curve required to reduce the incidence of surgical iatrogenic events, a novel device has been developed to emit real-time nerve auto-fluorescence in parallel to artificial intelligence (AI) surgical navigation software (SNS) feedback to isolate and accurately identify nerve structures during surgery. The authors herein present one of the first cases of a benign parotid tumor excision implementing dual AI and nerve auto-fluorescence technology for a minimally invasive, nerve-sparing parotidectomy. This report underscores the potential of nerve auto-fluorescence-guided surgery to improve surgical precision and patient outcomes.

## Introduction

The most common indication for parotidectomy is excision of a benign or malignant neoplasm, of which 75-80% are benign and of primary parotid origin [1]. The different types of parotid surgery include extracapsular dissection, partial/superficial parotidectomy, total parotidectomy, and radical parotidectomy [2,3]. All techniques require substantial awareness of anatomical landmarks for cautious surgical navigation to avoid inadvertent nerve damage. Contemporary performance of parotid surgery implies the risk of various complications, including but not limited to hematoma, transient/complete nerve paralysis, Frey syndrome, and first-bite syndrome [4,5]. With the development of superficial musculoaponeurotic system (SMAS) flaps and the routinary use of continuous electromyography, postoperative nerve damage has decreased [6]. Nevertheless, the incidence of transient paralysis ranges from 16.6% to 34%. Moreover, although 90% of patients recover within one month of surgery, paralysis can potentially last as long as 18 months [5,7]. The preservation of nerve integrity is critical for surgeries with anatomically complex regions, such as head and neck procedures, including parotidectomy. The traditional methods for nerve isolation and identification rely on anatomical landmarks and tactile feedback, all of which are insufficient regardless of the presence of challenging anatomy, scarring tissue, or neoplasm size effect [6]. To address this challenge, a novel nerve auto-fluorescence technology was developed to enable real-time visualization of nerves during surgery. The technology is further complemented by artificial intelligence (AI) surgical navigation software (SNS) that works in concomitance to tissue auto-fluorescence emission and conveys an additional layer of accuracy and anatomical mapping.

To the authors' best knowledge, this is the first case report illustrating the successful application of this technology in a patient undergoing parotidectomy for a benign tumor.

## Case presentation

This is the case of a 59-year-old female patient with a history of type 2 diabetes mellitus and hypertension, who presented for the clinical evaluation at Sanatorio Otamendi & Miroli (Buenos Aires, Argentina) of a left parotid-region mass eliciting facial deformity and no functional nerve impairment. Initial evaluation revealed a firm, well-defined, non-mobile, non-tender mass, at the level of the left mandibular angle with an approximate 3 cm span, which had progressively increased in size over the last four months prior to evaluation. Preoperative ultrasound performance and fine-needle aspiration biopsy revealed the presence of a solid, well-circumscribed benign neoplasm (MILAN 4A classification). The decision was made to perform an elective parotidectomy utilizing the Dendrite® (Gera, Germany) hand-held device for nerve auto-fluorescence visual aid, and Intelligent Surgical Eye® (ISE) SNS (Miami, FL) for surgical navigation aid of all relevant nerve structures. The patient was provided with verbal and written consent prior to intervention.

Surgical technique

**Figure 1:**
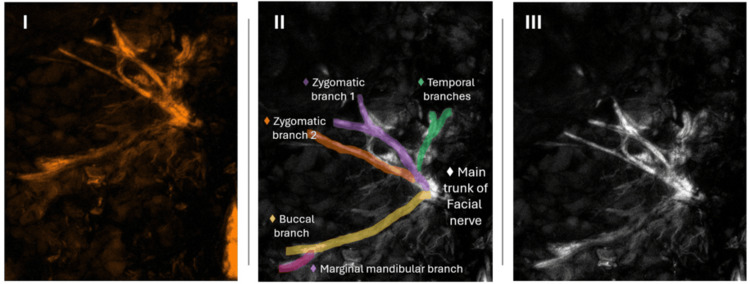
Nerve auto-fluorescence identification via the Dendrite® device and Intelligent Surgical Eye® (ISE) surgical navigation software (SNS) Images I and III showcase the overlay and contrast modes, respectively. Image II exemplifies the isolation of observable nerve anatomy and their projected anatomy.

**Figure 2:**
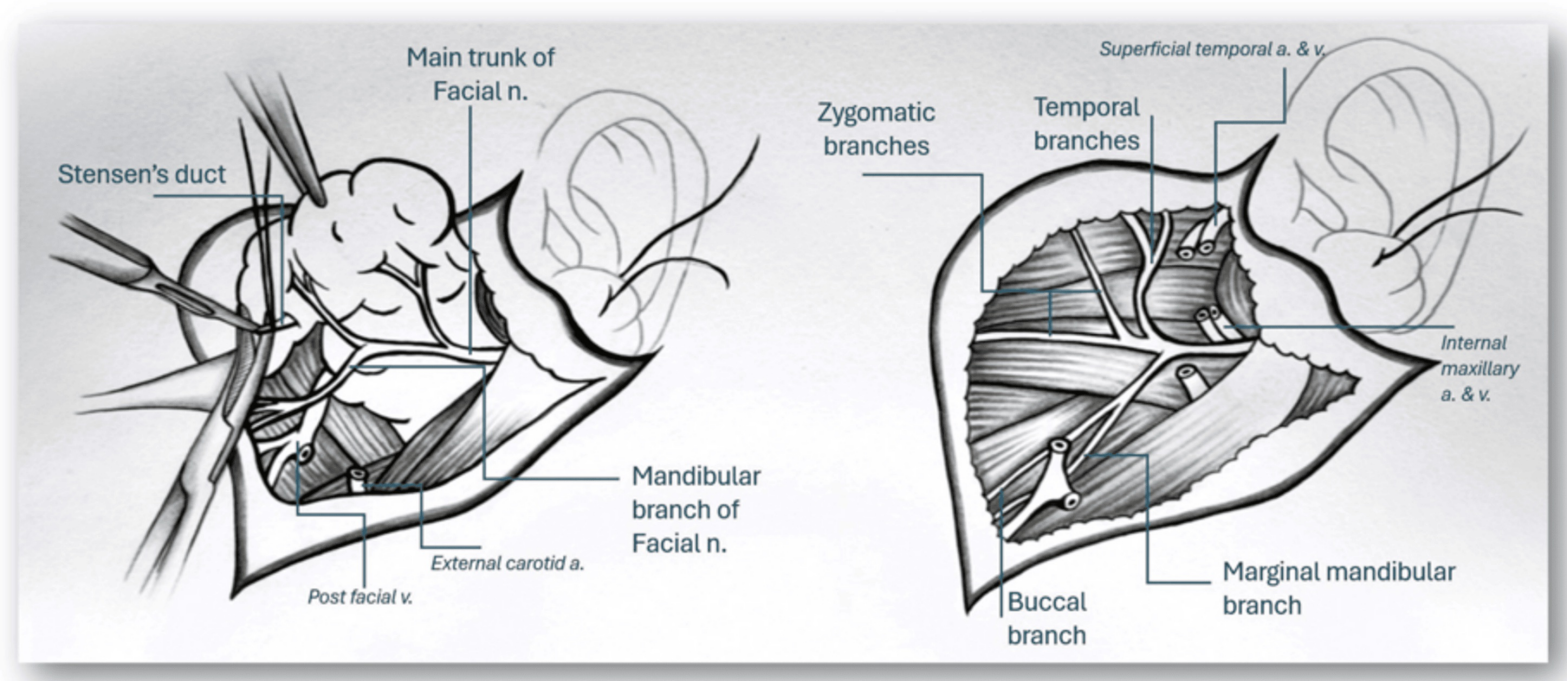
Surgical technique: parotidectomy Left: Dissection of the parotid gland and main trunk of the facial nerve. Right: Post parotidectomy identifying all relevant branches intact following dissection and resection. Abbreviations: a. - Artery. n. - Nerve. v. - Vein. Illustration by Rene Aleman MD.

The procedure was meticulously planned to achieve complete tumoral excision with preservation of the facial nerve and all neighboring nerve branches. Upon induction of general anesthesia, a Blair incision was carried out from the preauricular skin crease around the lobule posteriorly and extended inferiorly into the cervical skin crease. Following the incision of the dermis and the platysma, an anterior skin flap was raised in a plane between the SMAS and the superficial capsule of the parotid gland. The parotid tissue was cautiously separated from the subcutaneous layer, and the sternocleidomastoid muscle (SCM) was dissected to locate the posterior belly of the digastric muscle. At this time, the Dendrite® device is brought into the surgical field for visualization of the greater auricular nerve, which is often sectioned during standard parotidectomy. The facial nerve and its branches were also identified at this time. The device implements near-ultraviolet light to excite nerve tissue and emit auto-fluorescence visualized via a high-sensitive lens. The nerve auto-fluorescence imagery is accompanied by ISE® SNS as a confirmatory layer of target tissue identification and projected anatomical path. The tandem technology was utilized in two imaging modalities: contrast (black and white) and overlay (colored) modes. The former provided high-contrast visualization of the nerves, and the latter enhanced nerve localization with colored projected mapping of relevant nerves (Figures 1-2).

**Figure 3:**
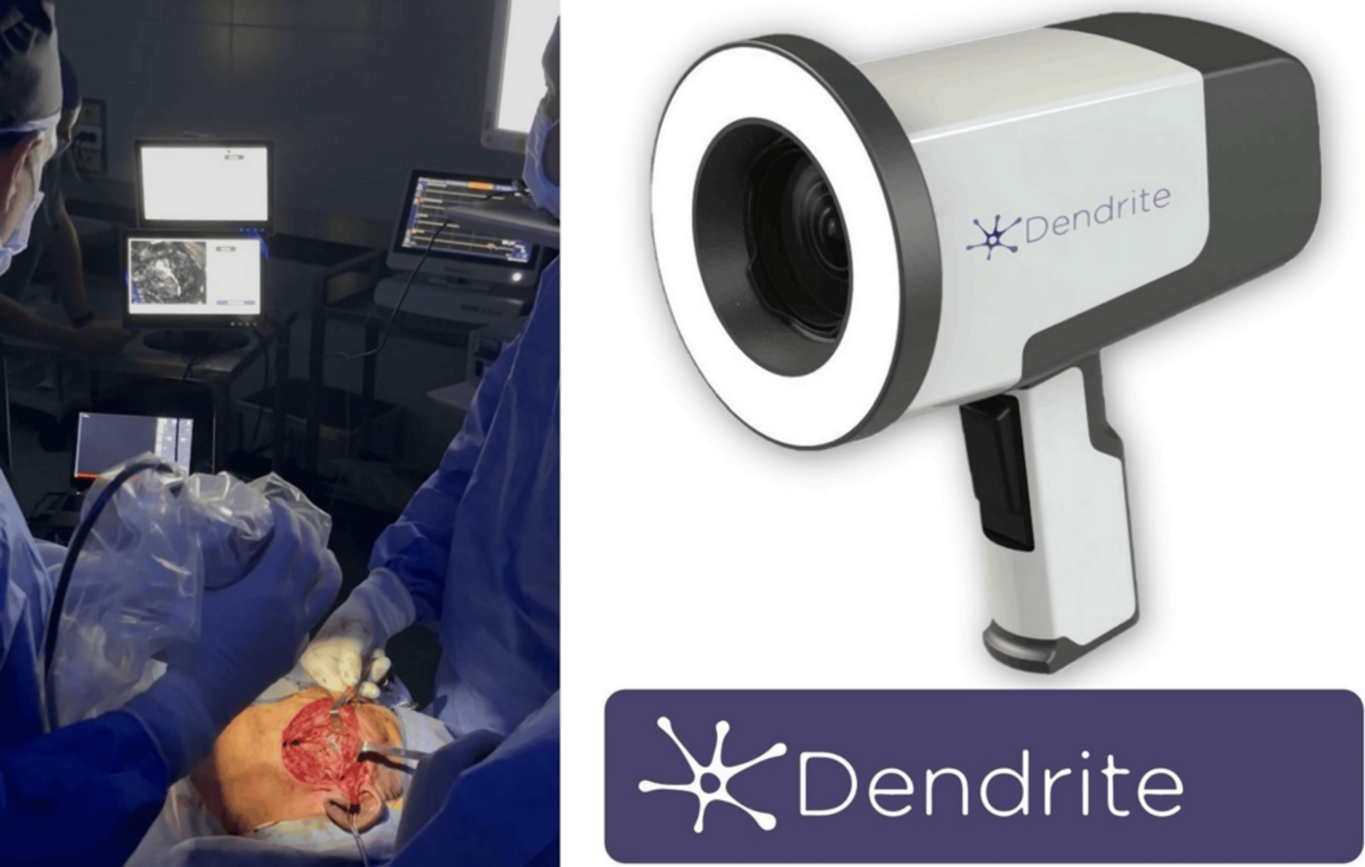
Intraoperative use of the handheld device Left: Intraoperative use of the handheld device to isolate the main trunk of the facial nerve and branches during parotidectomy. Right: Dendrite device dimensions

The procedure continues with the division of the attachments of the SCM and cartilage of the external auditory canal from the gland to allow for rotation and mobilization. The dissection of the facial nerve, its root, and all its branches is carried out under the direct guidance of the surgical device and SNS. When maneuvering within the parapharyngeal space, the external carotid, deep transverse facial, superficial temporal arteries, and the retromandibular and superficial temporal veins are ligated and divided to complete gland excision. The Stenson duct is identified and followed up towards the oral mucosa to avoid stone retention. The surgical site was polished for bleeding, thorough hemostasis was achieved, a drain was placed, and closure was performed in a standard multi-layer fashion to promote healing. Prior to closure, a final and confirmatory nerve auto-fluorescent evaluation was performed to ensure the integrity of the facial nerve and all its branches and confirm no interruption of normal anatomical trajectory. The procedure was completed with no complications, and the patient was transferred to the post-anesthesia care unit. Figure 3 showcases the intraoperative nerve identification during parotidectomy - for reference, Figure 1 is a detailed view of what is being shown during the procedure. 

Postoperative care

The patient tolerated the procedure well with no device- or procedural-associated complications. Nerve auto-fluorescent guidance enabled a precise identification and preservation of the facial nerve and its branches, including nerves <1 mm in diameter. The tumor was completely excised, and in standard fashion, postoperative evaluation showed no signs of nerve dysfunction or other common complications. The patient was discharged home 24 hours after surgery in stable condition and continued the normal course of recovery after four weeks of follow-up.

## Discussion

The correct identification and preservation of the facial nerve and its branches is the crucial step for successful parotid gland surgery. The oversight of nerve identification withholds significant complications that remain a concern despite current advances in intraoperative nerve integrity monitoring (NIM) and overall aggregate surgical experience. It is well known that head and neck surgery has a steep learning curve attributable to the challenging anatomical complexity innate to all types of interventions, rendering a superimposed risk variable for the existing common complications. The nerve injury complications of interest during parotid gland surgery are first-bite syndrome, Frey syndrome, and facial nerve transient/permanent paralysis. Altogether, their etiology stems from inadvertent nerve injury, and the prevention of their occurrence continues to be a top priority. Advances in fluorescent-guided surgery have paved the path to expanding the development and understanding of visual aid tools with the potential to decrease surgical learning curves requiring a high degree of anatomical landmarks awareness whilst providing feasibility, safety, cost-effectiveness, and clinical reproducibility. This case exemplifies the transformative potential of fluorescent imaging in enhancing surgical precision.

As indicated by the United Kingdom National Multidisciplinary Guidelines, the identification of the facial nerve during parotid gland surgery reduces the likelihood of iatrogenic injury [2]. Traditional methods, such as NIM and reliability of relevant neighboring anatomy, are limiting factors to achieving accuracy in the identification and preservation of all nerve structures, especially in cases involving anatomical variation or tumoral-induced distortion of tissular planes [8]. The inadequacy of current techniques results in the preoperative disclosure and counseling of all patients undergoing parotid surgery about the risks of temporary or permanent facial paralysis [9]. Moreover, contemporary practice describes the inevitable sectioning of the greater auricular nerve during initial incision and exposure of the gland and advocates for no repair of mid-facial branches when tumoral resection results in anatomical deformity [10,11]. Seemingly, these are inevitable surgical conducts taken secondary to the absence of tools to enhance surgical navigation and deliver accurate identification of primordial structures.

The technology herein described relies on the natural auto-fluorescent properties of central and peripheral nerves elicited as a result of near-ultraviolet light excitation. The emitted imagery feedback is filtered through a lens with an expanded range of light spectrum to capture all near-ultraviolet wavelengths emitted by the nerve’s autofluorescence. The outcome is the real-time, high-resolution imaging of nerves, including those <1 mm in diameter [12]. The conceptualization of the Dendrite® device was the result of modifying said proof of concept, which now has the full capability to perform during in-vivo settings [13]. Additionally, the dual modality (overlay and contrast modes) further aids in the accuracy of nerve localization. In parallel, the ISE® SNS is still under development owing to its dependency on in-vivo imagery data accrual to improve sensitivity and specificity capabilities in the mapping of nerve structures, including their projected trajectories. Nevertheless, the software continues to show promising results when implemented in clinical practice.

The successful outcome in this case highlights several advantages of this technology. Firstly, the improved safety is supported by real-time, non-invasive, nerve visualization, thus minimizing the risk of nerve injury and ensuring better functional outcomes. Secondly, the enhanced precision provides the ability to identify nerves with high levels of accuracy and facilitates thorough resections with the unequivocal preservation of critical structures. Lastly, the broad capability of this technology is evident as it has the potential to translate into other specialties, including breast surgery, genito-urinary surgery, and hernia repair surgery. Notably, this technique stands out from other fluorescent-guided surgery applications as it does not require the administration of any fluorescent dyes, thus facilitating its application in the operating room. The patient’s uneventful perioperative course requiring no additional clinical evaluation further contributes to the feasibility, safety, and efficacy of this technique. The authors, however, acknowledge the limitations of this report: its level of evidence and the lack of a larger sample size. However, the widespread adoption of this technology will give place to a solution to said limitations and provide validation of the benefits across diverse surgical scenarios.

## Conclusions

Nerve auto-fluorescence technology represents a significant advancement in surgical precision and safety. Enabling real-time visualization of nerves enhances surgical navigation, maneuverability, and dexterity, all while minimizing the potential risk of inadvertent nerve damage and associated complications. This case report illustrates the successful application of novel tandem technology in a patient undergoing parotidectomy, setting a promising precedent for comprehensive adoption in surgical practice for other specialties.
